# White matter microstructural differences in children and genetic risk for multiple sclerosis: A population-based study

**DOI:** 10.1177/13524585211034826

**Published:** 2021-08-11

**Authors:** C Louk de Mol, Rinze F Neuteboom, Philip R Jansen, Tonya White

**Affiliations:** Department of Neurology, MS Center ErasMS, Erasmus MC University Medical Center Rotterdam, Rotterdam, The Netherlands/The Generation R Study Group, Erasmus MC University Medical Center Rotterdam, Rotterdam, The Netherlands; Department of Neurology, MS Center ErasMS, Erasmus MC University Medical Center Rotterdam, Rotterdam, The Netherlands; The Generation R Study Group, Erasmus MC University Medical Center Rotterdam, Rotterdam, The Netherlands/Department of Complex Trait Genetics, Center for Neurogenomics and Cognitive Research, Amsterdam Neuroscience, Amsterdam UMC, Amsterdam, The Netherlands/Department of Clinical Genetics, Amsterdam UMC, Vrije Universiteit Amsterdam, Amsterdam, The Netherlands; Department of Child and Adolescent Psychiatry, Erasmus MC University Medical Center Rotterdam, Rotterdam, The Netherlands; Department of Radiology and Nuclear Medicine, Erasmus MC University Medical Center Rotterdam, Rotterdam, The Netherlands

**Keywords:** Multiple sclerosis, genetic association studies, white matter, epidemiology, child development

## Abstract

**Background:**

MS patients show abnormalities in white matter (WM) on brain imaging, with heterogeneity in the location of WM lesions. The “pothole” method can be applied to diffusion-weighted images to identify spatially distinct clusters of divergent brain WM microstructure.

**Objective:**

To investigate the association between genetic risk for MS and spatially independent clusters of decreased or increased fractional anisotropy (FA) in the brain. In addition, we studied sex- and age-related differences.

**Methods:**

3 Tesla diffusion tensor imaging (DTI) data were collected in 8- to 12-year-old children from a population-based study. Global and tract-based potholes (lower FA clusters) and molehills (higher FA clusters) were quantified in 3047 participants with usable DTI data. A polygenic risk score (PRS) for MS was calculated in genotyped individuals (*n* = 1087) and linear regression analyses assessed the relationship between the PRS and the number of potholes and molehills, correcting for multiple testing using the False Discovery Rate.

**Results:**

The number of molehills increased with age, potholes decreased with age, and fewer potholes were observed in girls during typical development. The MS-PRS was positively associated with the number of molehills (β = 0.9, SE = 0.29, *p* = 0.002). Molehills were found more often in the corpus callosum (β = 0.3, SE = 0.09, *p* = 0.0003).

**Conclusion:**

Genetic risk for MS is associated with spatially distinct clusters of increased FA during childhood brain development.

## Introduction

Multiple sclerosis (MS) is a severe demyelinating disease of the CNS involving the gray and white matter (WM) of the brain and spinal cord.^1^ While the exact pathogenesis of MS remains unclear, it is known that genetic factors contribute considerably to the pathogenesis of the disease. The largest genome-wide association study (GWAS) of MS to date has identified a large number of genome-wide significant and suggestive risk variants (mostly single nucleotide polymorphisms, SNPs) that contribute to the etiology of MS.^2^

To capture the polygenic effect of MS risk variants, polygenic risk scores (PRSs) can be calculated by combining additive effects of common variants across the genome. Most previous PRS studies have calculated the MS-PRS based on risk variants that reached genome-wide significance in the GWAS, while leaving out sub-threshold risk variants that convey additional genetic risk.^2–4^

MS patients have abnormalities in microstructural measures of several WM tracts compared to healthy individuals, including WM alterations measured using diffusion tensor imaging (DTI) with decreased fractional anisotropy (FA) and increased mean diffusivity, attributed to the loss of myelin and axonal degeneration during the disease process.^5^ We recently showed that a higher MS-PRS is related to a higher global FA in typically developing school-age children in a population-based study.^6^ However, the spatial characteristics of the underlying pathophysiology of higher global FA related to a higher genetic risk for MS in our earlier study could not be investigated due to the different assumptions of different imaging analysis approaches. Voxel-based DTI analyses require that the WM differences or lesions on brain imaging are spatially overlapping. Using probabilistic tractography, an alternative algorithm for analyzing DTI images that extracts tract-based WM measures, we only assessed global WM metrics along an entire tract. These algorithms thus are not optimal for capturing spatially heterogeneous WM abnormalities that occur in MS.^7^

Relatives of MS patients show increased WM hyperintensities, but no differences in overall WM integrity.^8,9^ Thus, it is possible that small clusters of microstructural abnormalities occur in some WM tracts in children with high genetic risk for MS. Imaging methods that assess WM microstructure without the assumption of overlap in the location of the WM abnormalities can provide a more accurate characterization into how genetic risk for MS affects neurodevelopment.

One approach to identify non-spatially-overlapping WM abnormalities in the brain is the “pothole” method.^10^ Potholes are clusters of contiguous voxels in which all voxels in the cluster are at least two standard deviations (SD) below the voxel-wise mean.^10^ Alternatively, clusters that are at least two SD above the mean are termed molehills. This method may yield new insights into early MS pathophysiology by investigating whether clusters of microstructural WM abnormalities, due to genetic risk for MS, are already present at an early age, when applied in a population sample of children.

Within this backdrop, we here study the association between an MS-PRS and non-spatially overlapping clusters of WM microstructural characteristics in school-age children across different study samples. Based on adult studies that show WM hyperintensities in healthy relatives of MS patients without global WM differences,^8,9^ we hypothesize that children at a high polygenic risk for MS will have more clusters of abnormal WM, as measured by a higher number of potholes and compensatory molehills.

## Methods

### Participant selection

We included participants from the imaging cohort of the Generation R study, a prospective population-based birth cohort.^11^ Between the ages of 8 and 12 years, 3992 children underwent magnetic resonance (MR) scanning on a study-dedicated research MR scanner.^11^ Participants included those with diffusion tensor imaging (DTI) data that passed quality control without incidental findings that would influence image processing (*n* = 3047). First, we assessed age and sex differences related to the presence of WM potholes and molehills. Subsequently, we selected unrelated participants of genetic European ancestry with good-quality genotype data available to investigate the association between genetic risk for MS and the number and size of potholes and molehills (*n* = 1087).

In addition, we identified non-overlapping participants from an earlier neuroimaging wave of the Generation R study as a replication sample (*n* = 185), who were scanned between the ages of 6 and 10 years old.^12^

The Generation R Study has been approved by the Medical Ethical Committee of the Erasmus Medical Center and is carried out according to the Declaration of Helsinki. The legal representatives of the children provided written informed consent for participation.

### Neuroimaging

Diffusion weighted images were collected on a single-study-dedicated 3 Tesla MR750w Discovery MRI scanner (General Electric, Milwaukee, WI, USA). The scan protocol, imaging procedures, and processing of the collected images have been described in earlier work from our imaging group.^11,13^ Briefly, DTI images were collected using an axial spin echo, echo planar imaging (EPI) sequence with 3 *b* = 0 scans and 35 diffusion directions with b = 900 s/mm^2^. The diffusion-weighted sequence was collected using the following parameters: *T*_R_ = 12,500 ms, *T*_E_ = 72 ms, field of view = 240 × 240, acquisition matrix = 120 × 120, number of slices = 65, slice thickness = 2 mm, asset acceleration factor = 2, frequency encoding direction = R/L, phase encoding direction = P/A.

Participants from the replication sample were scanned on a different GE MR platform (GE 3 T MR750 Discovery System, Milwaukee, Wisconsin) with similar sequence parameters, described elsewhere.^12^

### Image processing

Imaging data were processed using a combination of FSL’s Brain’s Software Library (FMRIB)^14^ and an in-house Python program. The diffusion-weighted images were first adjusted for motion and eddy-current artifacts using the FSL “eddy_correct” tool.^15^ Transformation matrices were extracted and used to rotate the gradient direction table to account for rotations applied to the image data.^16^ Non-brain tissue was removed using FSL’s Brain Extraction Tool.^17^ The diffusion tensor model was fit using RESTORE implemented in Camino,^18^ resulting in FA scalar maps. FA scalar images were converted into MNI space using the first three steps of FSL’s Tract Based Spatial Statistics non-linear registration.^19^

### Pothole and molehill assessment

The quantification and mapping method of the potholes has been previously described by White et al.^10^ To summarize, the FA images in standard space were used to derive a mean and SD image for each voxel using all subjects with both good-quality data and good registration to standard space.^10^ Using the mean FA and SD images, a voxel-wise *z*-image was calculated for every participant. Only voxels with a group mean FA greater than 0.2 were used in constructing the *z*-transformed FA images.^10,20^ Considering the size heterogeneity in MS WM abnormalities, these *z*-images were used to quantify different sizes of clusters of voxels (clusters of 25, 50, 100, and 200 mm^3^ contiguous voxels) that were either below (*z* < –2.0: pothole) or above two SD (*z* > 2.0: molehill). To localize the potholes and molehills, the number of WM anomalies was quantified within the WM tracts defined by the Johns Hopkins University White Matter Atlas.^21^ The global and tract-based number of potholes and molehills were used in the statistical analyses.

### Genetic data

Blood samples were extracted either from cord blood at birth or via a venipuncture during a visit to the research center. Genetic data were extracted using either a Illumina 610 K or 660 K SNP array (Illumina, San Diego, CA, USA).^22^ Quality control procedures, including imputation of the genotype data and calculation of principal components (PCs), have been described in previous work.^23^ To summarize, we selected participants of European ancestry based on the first four PCs inside the range of the HapMap Phase II Northwestern European founder population.^24^ Data from the 1000 Genomes (Phase I version 3) project were used to impute our genotype data and calculate the PRS.^25^

Calculation of the MS-PRS has been described previously.^6^ We used the largest discovery GWAS of MS to date (*n* = 41,505 participants; 14,802 cases/26,703 controls), carried out by the International Multiple Sclerosis Genetics Consortium (IMSGC).^2^ The PRS was calculated using PRSice 2 software, and SNPs located in high linkage disequilibrium regions were removed.^26^ Multiple *p*-value thresholds were used for the inclusion of SNPs in the PRS; however, in the current study, we only used the PRS with a *p*-value threshold (*P*_T_) of less than 0.01, since this PRS was most strongly associated with DTI measures in our previous work.^6^ We used rs3135388 as tag SNP to reflect the HLA-DRB1*15:01 haplotype.^27^

### Statistical analysis

All analyses were performed using the statistical software package R (version 3.6.1).^28^ For non-response analyses, we tested for differences in descriptive characteristics and genetic MS risk between the study sample and children within the Generation R cohort who did not participate in the DTI study using two-sided *t*-tests and chi-square tests. To assess the number and location of potholes/molehills associated with sex, age, and the MS-PRS, we performed multiple linear regression. We performed additional sensitivity analyses for age and sex adjusting for maternal education and ethnicity. The analyses involving genetic risk were adjusted for age at scan, sex, and the first 10 genetic PCs. Subsequently, we performed sensitivity analyses adjusting for the level of maternal education. Tract-based numbers of potholes/molehills were used post hoc to identify local differences if a global effect was observed, with an adjustment for handedness in lateralized tracts. Multiple linear regression analyses were used to replicate significant associations in the replication sample.

We used the False Discovery Rate (FDR) to correct for multiple testing on the total number of statistical tests when investigating the associations with descriptive statistics (16 tests) and genetic risk for MS (8 tests).^29^ When investigating exploratory post hoc tract-specific associations FDR was performed for all the tracts involved (17 tests).

## Results

### Sample information

A total of 3047 participants had usable DTI data available for investigating age- and sex-related differences in potholes and molehills. This sample had a median age of 9.94 years (interquartile range (IQR): 9.77–10.32) and sex was split evenly (49.7% male) (Table 1). Compared to Generation R participants who did not participate in the DTI study (*n* = 6853), the participants included were more often Dutch (*p* < 0.001) and were of higher maternal education (*p* < 0.001). No difference was found in sex between the two samples (*p* = 0.18).

**Table 1.** Descriptive characteristics of the samples used in this study.

	Total study sample (*n* = 3047)	Genetic and DTI data available (*n* = 1087)	Replication sample (*n* = 185)	*p*-value
Age in years, median (IQR)	9.94 (9.77–10.32)	9.96 (9.78–10.36)	8.51 (7.55–9.03)	< 0.001
Male, *n* (%)	1513 (49.7)	551 (50.7)	100 (54.1)	0.46
Level of maternal education, *n* (%)				< 0.001
Low	182 (6.0)	10 (0.9)	1 (0.5)	
Middle	1117 (36.7)	317 (29.2)	72 (38.9)	
High	1506 (49.4)	739 (68.0)	110 (59.5)	
Unknown	242 (7.9)	21 (1.9)	2 (1.1)	
Reported ethnicity, *n* (%)				< 0.001
Dutch	1871 (61.4)	980 (90.2)	176 (95.1)	
Western	269 (8.8)	83 (7.6)	7 (3.8)	
Non-western	852 (27.9)	24 (2.2)	2 (1.1)	
Unknown	55 (1.8)	0 (0.0)	0 (0.0)	

DTI: diffusion tensor imaging; IQR: interquartile range. Analyses of variance (ANOVA) and chi-square tests were applied to test for differences between the study samples.

After selecting for availability and quality of genotype data, European ancestry, and relatedness, 1087 participants remained for the PRS analyses. These participants had a median age of 9.96 years (IQR: 9.78–10.36), with an even distribution of sex (50.7% male) (Table 1). We found no differences in sex (*p* = 0.63) and mean MS-PRS (*p* = 0.97) of this sample compared to European genotyped participants who did not participate in the DTI study (*n* = 1743). However, we again observed a larger proportion of higher maternal education compared to the non-included participants (*p* < 0.001).

In total, 185 non-overlapping participants were identified from an earlier Generation R neuroimaging wave as a replication sample. These participants were significantly younger compared to our main study sample (median age: 8.51 years, *p* < 0.001), but the distribution of sex was comparable (54.1% male, *p* = 0.46) (Table 1).

### White matter characteristics

**Figure 1.**
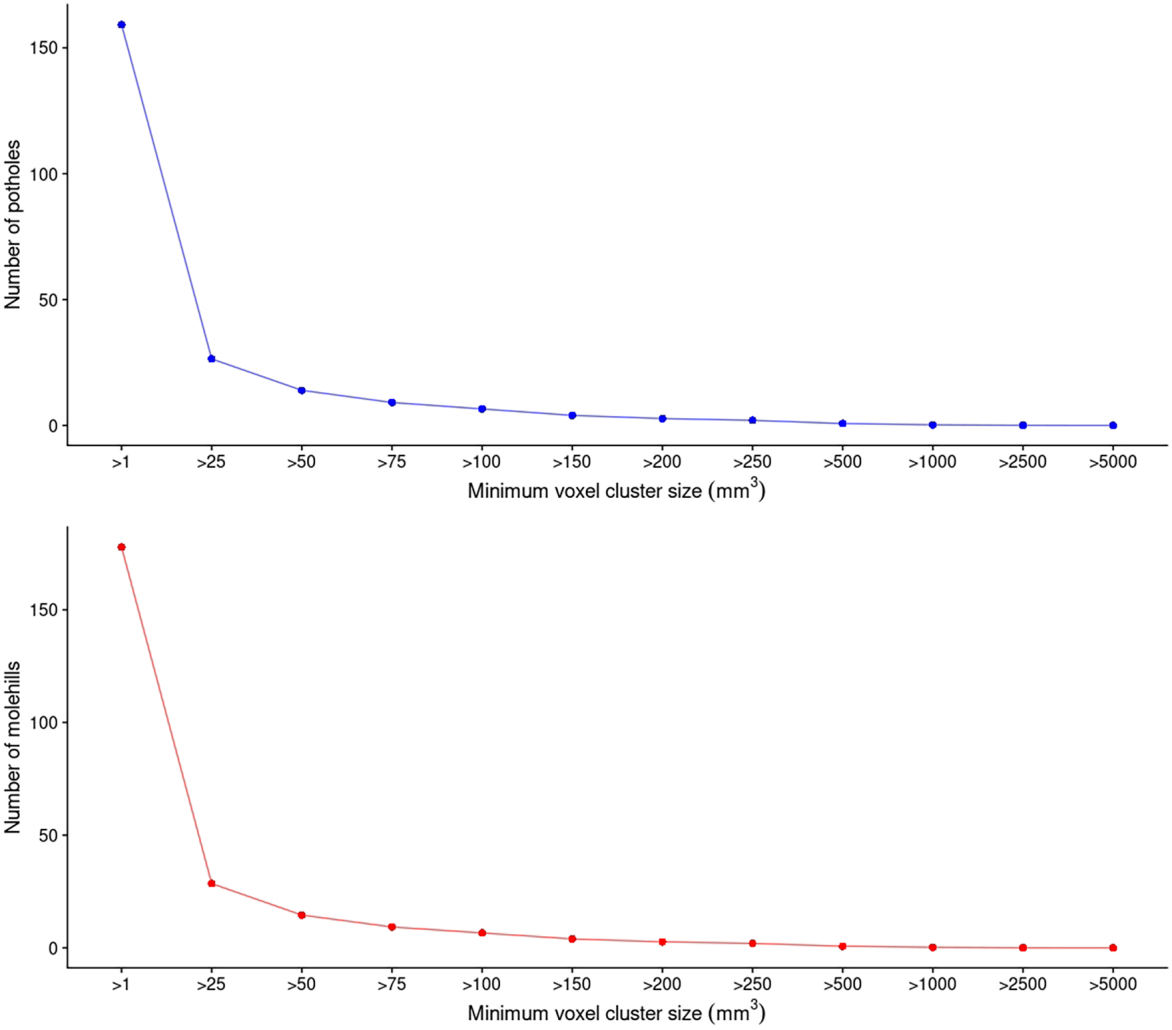
Mean number of potholes and molehills across different voxel cluster sizes (mm^3^) (*n* = 3047).

**Figure 2.**
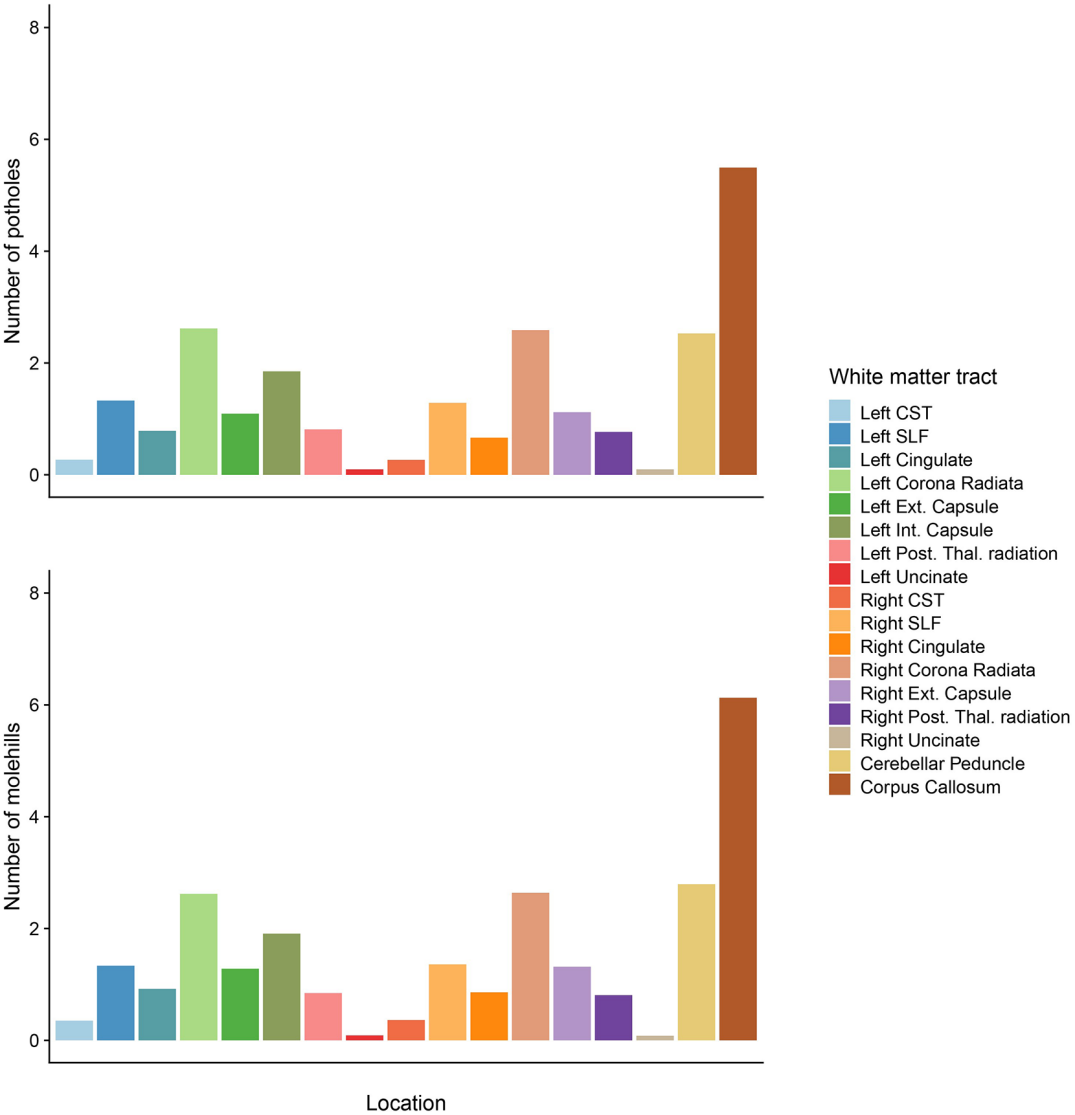
Mean number of potholes and molehills per white matter tract across all subjects (*n* = 3047).CST: corticospinal tract; Ext: external; Int: internal; Post: posterior; SLF: superior longitudinal fasciculus; Thal: thalamic.

Figure 1 shows the distribution of potholes and molehills across the different cluster sizes in the 3047 participants. Given a voxel cluster size of > 25 mm^3^, we observed a median of 23 potholes (IQR: 14–36) and 26 molehills (IQR: 15–39). Figure 2 shows the spatial distribution of potholes and molehills across 17 WM tracts (voxel cluster size of > 25 mm^3^).

**Figure 3.**
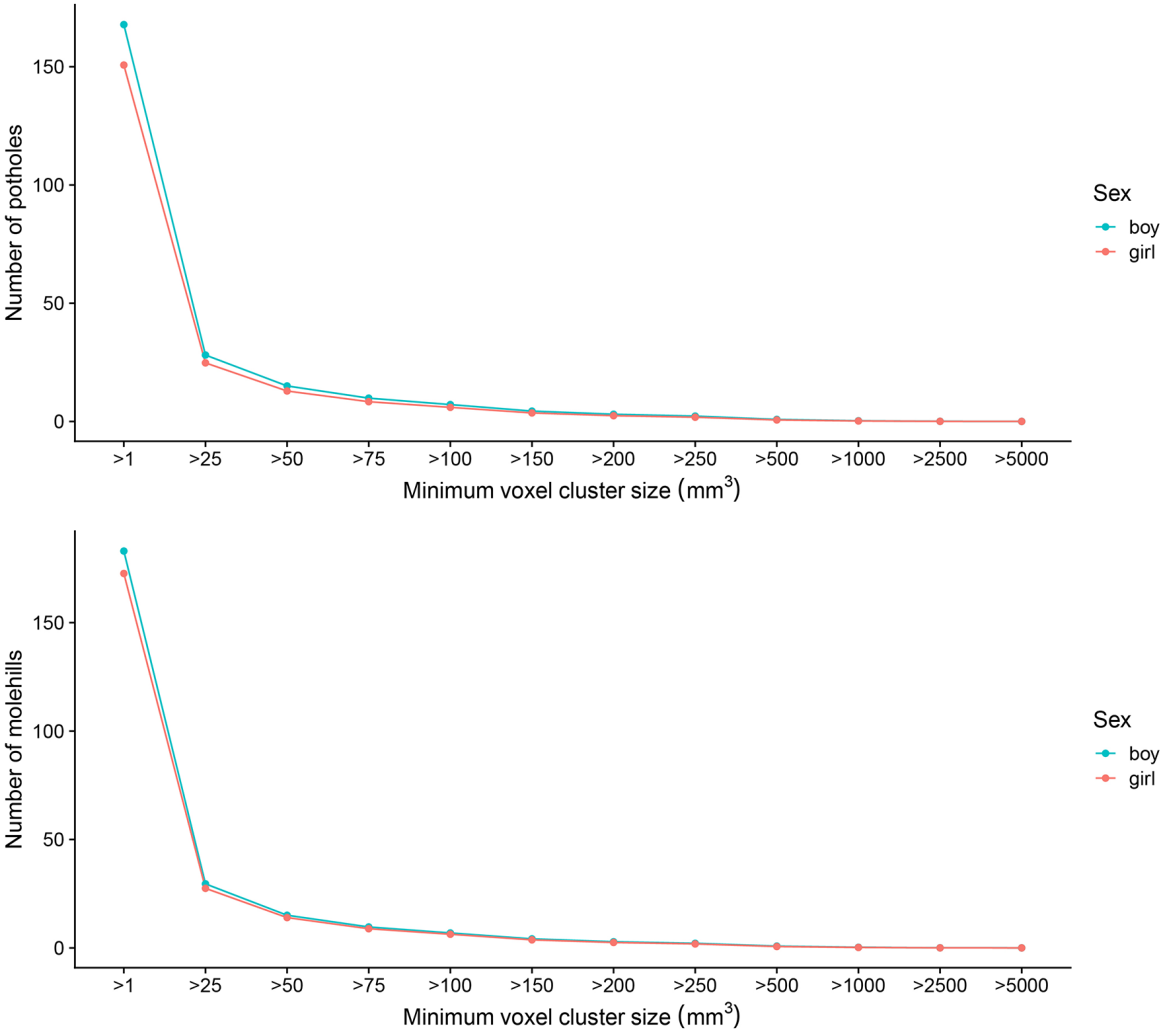
Mean number of potholes and molehills across different sexes per different voxel cluster sizes (mm^3^) (*n* = 3047).

Sex and age showed the strongest association with a voxel cluster size of 25 mm^3^ for both potholes and molehills. Girls were found to have significantly less potholes (β = −3.6, standard error (SE) = 0.57, *p* = 3.09 × 10^−10^) and molehills (β = −1.7, SE = 0.59, *p* = 3.06 × 10^−3^) compared to boys, independent of age at scan (Figure 3, Table 2). After adjustment for intracranial volume, girls continued to have significantly less potholes, but more molehills were observed compared to boys (β = 2.8, SE = 0.66, *p* = 2.50 × 10^−5^). These differences remained significant for both potholes and molehills after additionally adjusting for reported ethnicity and maternal education (*p* = 7.77 × 10^−4^ and *p* = 3.49 × 10^−5^, respectively).

**Table 2.** Effects of sex and age on the number of potholes and molehills (*n* = 3047).

Outcome, minimum voxel cluster size in mm^3^	Female sex					Age in years				
	β	SE	Δ*R*^2^	*p*	FDR	β	SE	Δ*R*^2^	*p*	FDR
Potholes										
25	−3.6	0.57	0.0127	3.09 ×10^−10^	**7.95 × 10** ^−10^	−3.5	0.47	0.0171	3.31 × 10^−13^	**1.79 × 10** ^−12^
50	−2.3	0.37	0.0127	3.48 × 10^−10^	**7.95 × 10** ^−10^	−2.1	0.30	0.0148	1.18 × 10^−11^	**3.78 × 10** ^−11^
100	−1.3	0.22	0.0104	1.33 × 10^−8^	**2.36 × 10** ^−8^	−1.1	0.18	0.0124	6.44 × 10^−10^	**1.29 × 10** ^−9^
200	−0.6	0.12	0.0091	1.11 × 10^−7^	**1.61 × 10** ^−7^	−0.5	0.10	0.0087	2.20 × 10^−7^	**2.94 × 10** ^−7^
Molehills										
25	−1.7	0.59	0.0028	3.06 × 10^−3^	**3.77 × 10** ^−3^	4.1	0.49	0.0229	4.06 × 10^−17^	**6.50 × 10** ^−16^
50	−0.9	0.36	0.0021	1.10 × 10^−2^	**1.17 × 10** ^−2^	2.2	0.30	0.0172	3.36 × 10^−13^	**1.79 × 10** ^−12^
100	−0.5	0.20	0.0020	1.32 × 10^−2^	**1.32 × 10** ^−2^	1.2	0.17	0.0169	5.53 × 10^−13^	**2.21 × 10** ^−12^
200	−0.3	0.11	0.0023	7.88 × 10^−3^	**9.01 × 10** ^−3^	0.5	0.09	0.0103	1.87 × 10^−12^	**2.98 × 10** ^−8^

FDR: False Discovery Rate; SE: standard error. Included: *n* = 3047 children, results were obtained by using multiple regression. Sex-specific effects were adjusted for age and age-specific effects were adjusted for sex. Significant values after FDR multiple testing correction are highlighted in bold.

**Figure 4.**
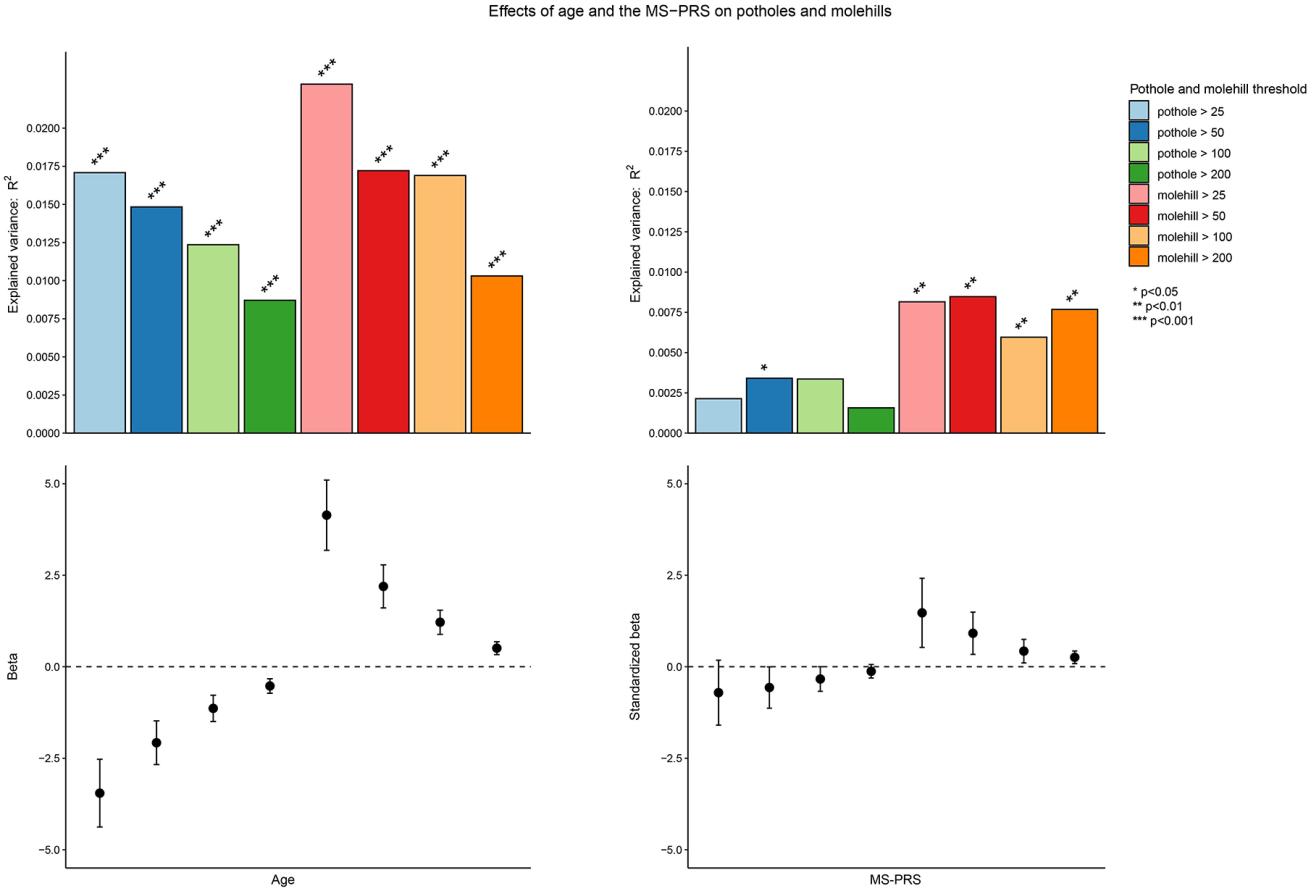
Associations of the MS-PRS and age with potholes and molehills across different voxel cluster sizes. MS: multiple sclerosis; PRS: polygenic risk score.

The number of potholes was, independent of sex, negatively associated with age (β = −3.5, SE = 0.47, *p* = 3.31 × 10^−13^) (Figure 4, Table 2), while the number of molehills showed a significant positive association with age (β = 4.1, SE = 0.49, *p* = 4.06 × 10^−17^). After additional adjustment for maternal education and ethnicity, these findings remained significant (*p* = 8.46 × 10^−13^ and *p* = 3.06 × 10^−15^, respectively).

Supplementary Table 1 shows the associations of age and sex with potholes and molehills mapped to different WM tracts.

### Polygenic MS risk

The MS-PRS (P_T_ < 0.01) showed a positive association with molehills across all voxel cluster sizes (Figure 4); however, the strongest association was observed with a voxel cluster size of > 50 mm^3^ (scaled β = 0.9, SE = 0.29, *p* = 0.002). These associations remained significant after multiple testing correction (Table 3). Negative associations were observed between the MS-PRS and the number of potholes; however, these did not pass multiple testing correction. The positive association between the MS-PRS and molehills remained significant after additional adjustment for the level of maternal education (*p* = 0.003).

**Table 3.** Effects of the MS-PRS (P_T_ < 0.01) on the number of potholes and molehills. (*n* = 1087).

Outcome, minimum voxel cluster size in mm^3^	MS-PRS (P_T_ < 0.01)				
	β	SE	Δ*R*^2^	*p*	FDR
Potholes					
25	−0.7	0.45	0.0021	1.18×10^−1^	1.35 × 10^−1^
50	−0.6	0.29	0.0034	4.90×10^−2^	6.84 × 10^−2^
100	−0.3	0.17	0.0034	5.13×10^−2^	6.84 × 10^−2^
200	−0.1	0.09	0.0016	1.86×10^−1^	1.86 × 10^−1^
Molehills					
25	1.5	0.48	0.0082	2.29×10^−3^	**9.15 × 10** ^−3^
50	0.9	0.29	0.0085	1.97×10^−3^	**9.15 × 10** ^−3^
100	0.4	0.16	0.0060	9.79×10^−3^	**1.96 × 10** ^−2^
200	0.3	0.09	0.0077	3.53×10^−3^	**9.43 × 10** ^−3^

FDR: False Discovery Rate; MS: multiple sclerosis; PRS: polygenic risk score; SE: standard error. Included: *n* = 1087 children, data are corrected for age, sex, and 10 genetic principal components (PCs). Significant values after FDR multiple testing correction are highlighted in bold.

Exclusion of rs3135388, the tag variant of HLA-DRB1*15:01, attenuated the association (scaled β = 0.9, SE = 0.30, *p* = 0.003). We observed an even stronger attenuation when excluding the entire major histocompatibility complex (MHC) region (scaled β = 0.8, SE = 0.29, *p* = 0.005); however, both associations remained significant.

In our exploratory analysis assessing molehills mapped to different WM tracts, we found a positive association between the MS-PRS and molehills located in the corpus callosum (scaled β = 0.3, SE = 0.09, *p* = 0.0003) (Supplementary Table 2). No association was observed between the MS-PRS and the overall size of potholes and molehills (Supplementary Table 3).

**Figure 5.**
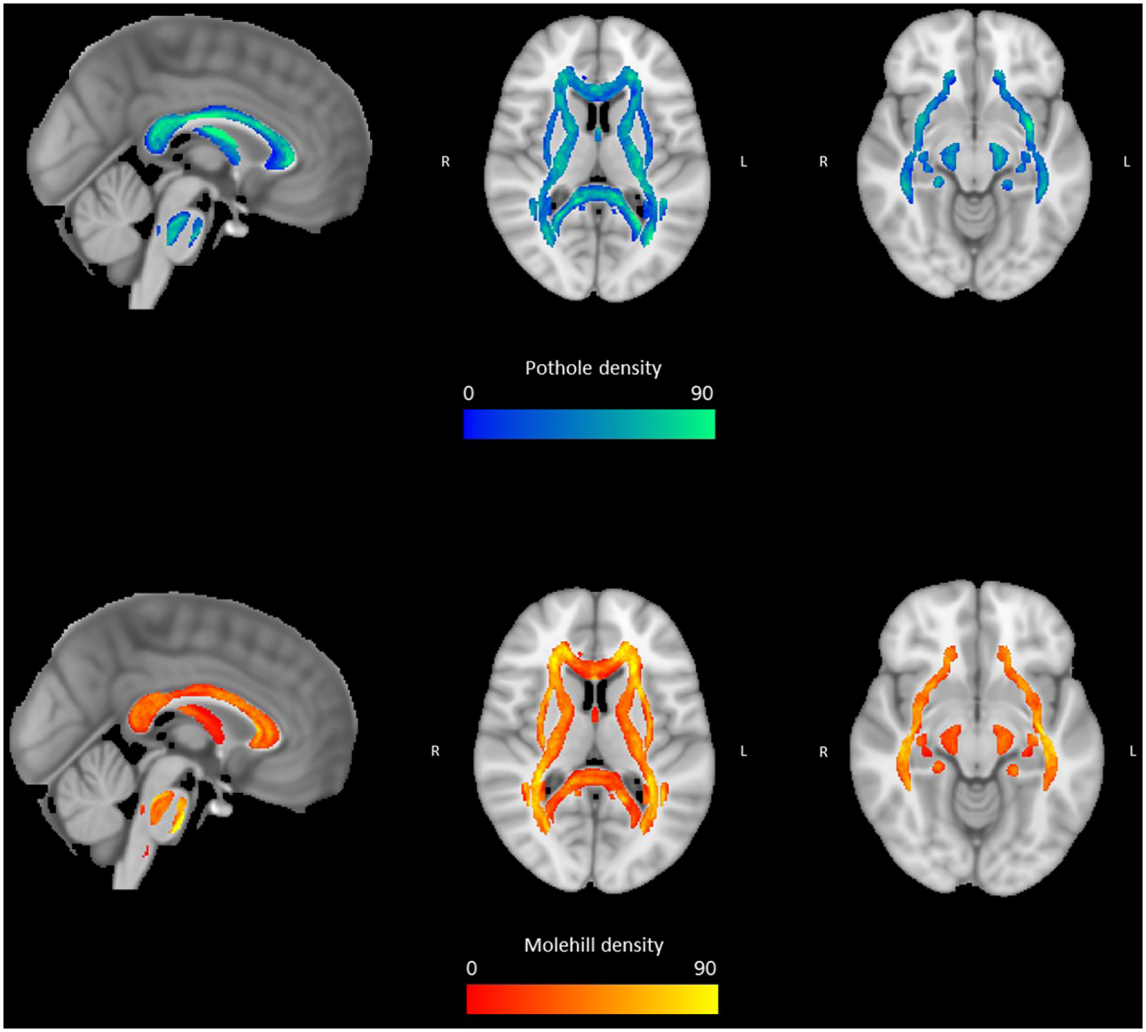
The spatial distribution of potholes and molehills in the study population at a voxel cluster size of 50 mm^3^. Green/blue represents the number and location of potholes and yellow/orange represents the number and location of molehills. From left to right: sagittal view, axial slice *z* = 10 and axial slice z = -10.

Figure 5 shows the spatial distribution of the potholes and molehills across all subjects at a voxel cluster size of 50 mm^3^.

### Replication sample

For a voxel cluster size of > 25 mm^3^, participants in the replication sample showed a median of 19 potholes (IQR: 11–30) and 25 molehills (IQR: 16–39). We observed similar significant associations when investigating the effects of age on potholes (β = −2.4, SE = 1.1, *p* = 0.03) and molehills (β = 2.7, SE = 1.3, *p* = 0.04), while sex showed non-significant effects.

The positive associations between the MS-PRS and the global number of molehills across different voxel cluster sizes were replicated in the independent sample (scaled β = 1.9, SE = 0.83, *p* = 0.03) (Supplementary Table 4). However, we observed no significant tract-specific associations of the MS-PRS in the replication sample (Supplementary Table 5).

## Discussion

We found that a higher genetic risk for MS is associated with a higher number of spatially independent clusters of elevated FA in 9- to 11-year-old children distributed globally throughout the brain, and more focused in the corpus callosum. In addition, we report age and sex related differences in both WM potholes and molehills in children from the general population.

WM molehills were positively associated with age, while potholes showed a negative association. This inverse correlation between age and potholes has been observed before.^10^ This is likely due to continued WM maturation during childhood.^30^ Since our method does not require WM abnormalities to be spatially overlapping, more molehills and less potholes suggest that WM matures in a regional, or even a more localized approach.

Girls have been shown to have earlier WM development compared to boys.^31^ These developmental differences could lead to the sexually dimorphic associations we observed in our study for the pothole and molehill WM measurements. After adjusting for total brain volume, girls had fewer potholes and more molehills, indicating greater maturation of WM development compared to males of the same age.

In our previous work, we report a higher global FA in children with higher genetic risk for MS.^6^ Our finding of more molehills in children with a higher MS-PRS offers greater precision to this finding. Relatives of MS patients show increased WM lesions compared to controls, which can be regarded as clusters of lower FA.^9^ Interestingly, relatives did not show differences in global WM microstructure, suggesting only the presence of small, focal lesions.^8^ By analyzing only global DTI measures in our earlier study we might have been unable to detect possible clusters that could reflect the early phase of MS WM abnormalities as a result of genetic factors. Compensatory mechanisms, as seen in other neurodegenerative diseases,^32^ and repair mechanisms as a response in other parts of the WM at this young age could have led to the higher global FA reported.^33^ However, our current study shows that genetic MS risk is not associated with clusters of lower FA (potholes) at this age, making a hypothesis of neurodegeneration early in life due to genetic risk for MS less likely. We do, however, show that genetic risk for MS is not only associated with global FA alterations, but also with non-spatially overlapping clusters of higher FA. As opposed to our earlier study, the pothole method allowed us to identify not only non-spatial overlapping alterations in WM, especially important for studying genetic risk for MS, but also regional associations of the MS-PRS with molehills in the corpus callosum. The spatial independence of these clusters and their increased localization in the corpus callosum is in line with the heterogeneity and location of WM alterations in MS.^7^ This association was not replicated in our replication sample, which may be a result of the smaller sample size and thus less power.

Patients with MS typically show lower FA and higher diffusivity attributed to WM degeneration.^5^ This may make our results seem counterintuitive. Yet, previous adult population studies investigating genetic risk for MS and WM characteristics suggest similar positive associations between genetic MS risk and FA.^34,35^ Increased presence of clusters with a higher FA could be due to the differences in myelin consistency^33^ or tighter neuronal clustering in individuals with higher genetic MS risk. Furthermore, our results may indicate accelerated or altered brain maturation in children with a higher MS-PRS, resulting in a higher FA and more molehills compared to children with low genetic risk. In addition, a higher FA and an increased number of molehills could indicate a lower number of crossing fibers in clusters across the brain in children with a higher MS-PRS.^36^ Finally, since most children with a higher MS-PRS will not develop MS, it is possible that a higher FA is a brain mechanism used to provide genetically driven protection or resilience against environmental factors associated with the later emergence of MS. Future studies are needed to include environmental risk factors for MS into the analyses to see how these key risk factors affect WM characteristics and interact with genetic MS risk in children.

There are a number of limitations to our study. First, WM alterations were investigated using a cross-sectional design, which does not allow investigating longitudinal effects of genetic risk for MS. Longitudinal studies can explore how MS risk factors alter WM development across the life-span and to see whether certain individuals show an MS-like phenotype from childhood, through adolescence, and into adulthood. Second, the GWAS for MS used to calculate the MS-PRS is relatively small compared to other disease-related GWAS studies. The lower sample size translates into less precision to identify important genetic risk variants. Third, since our longitudinal study was initiated over 8 years ago, to offer the opportunity to assess absolute change in MR parameters we are maintaining the same sequence and 8-channel head coil. However, MR upgrades, the use of more optimal sequences (i.e. multiband) and a 32-channel head coil may provide better signal-to-noise ratios. In addition, the single-shell acquisition and the low number of diffusion directions used in our diffusion MRI sequence limits us from performing more recent diffusion analyses methods (e.g. neurite orientation dispersion and density imaging and constrained spherical deconvolution). Because the “pothole” method makes use of DTI data, we have limitations in investigating the role of genetic MS risk in more fine-grained properties of brain WM, compared to these newer multi-shell diffusion analyses methods. Finally, the EPI sequence collected in our study was designed to limit loss due to distortion, however no additional field map was used for distortion correction.

In spite of the limitations, there are also a number of strengths of our study. First, using a large population-based sample of developing children can provide crucial insights into the underlying pathophysiology associated with genetic risk for MS before possible disease onset. Second, the extensive data collection of the Generation R Study allows for the adjustment for important confounders. Third, the children were included prospectively and all scanned on the same study-dedicated scanner, eliminating possible inter-scanner differences. Finally, children were included in a narrow age-range, minimizing the effect of age-related differences on our MS-PRS-related outcomes.

To conclude, we observed that higher genetic risk for MS causes non-spatially overlapping clusters of WM alterations in the developing brain of school-age children. This suggests that MS risk variants affect the white matter of the brain at an early age in children from the general population, which may subsequently lead to an increased vulnerability for MS pathophysiology.

## Supplemental Material

sj-docx-2-msj-10.1177_13524585211034826 – Supplemental material for White matter microstructural differences in children and genetic risk for multiple sclerosis: A population-based study Click here for additional data file. Supplemental material, sj-docx-2-msj-10.1177_13524585211034826 for White matter microstructural differences in children and genetic risk for multiple sclerosis: A population-based study by C Louk de Mol, Rinze F Neuteboom, Philip R Jansen and Tonya White in Multiple Sclerosis Journal

sj-pdf-1-msj-10.1177_13524585211034826 – Supplemental material for White matter microstructural differences in children and genetic risk for multiple sclerosis: A population-based study Click here for additional data file. Supplemental material, sj-pdf-1-msj-10.1177_13524585211034826 for White matter microstructural differences in children and genetic risk for multiple sclerosis: A population-based study by C Louk de Mol, Rinze F Neuteboom, Philip R Jansen and Tonya White in Multiple Sclerosis Journal
