# Supplementary material for: White matter microstructural differences in children and genetic risk for multiple sclerosis: A population-based study
Source: Mult Scler. 2021 Aug 11;28(5):730–41. doi: 10.1177/13524585211034826 (PMC8978478; doi:10.1177/13524585211034826)
Supplement: sj-docx-2-msj-10.1177_13524585211034826 – Supplemental material for White matter microstructural differences in children and genetic risk for multiple sclerosis: A population-based study [file sj-docx-2-msj-10.1177_13524585211034826.docx]

**Supplementary Table 1: Effects of the age and sex on the number of potholes and molehills located in specific WM tracts. (n=3,047)**

|  | **Female sex** | | | | | **Age in years** | | | | |
| --- | --- | --- | --- | --- | --- | --- | --- | --- | --- | --- |
| **Outcome, WM tract** | **β** | **SE** | **ΔR^2^** | **p** | **FDR** | **β** | **SE** | **ΔR^2^** | **p** | **FDR** |
| **Potholes** |  |  |  |  |  |  |  |  |  |  |
| Cerebellar peduncle | -0.20 | 0.07 | 0.0027 | 3.94×10^-3^ | **0.01** | -0.03 | 0.05 | 1.33×10^-4^ | 0.52 | 0.65 |
| Corpus callosum | -0.14 | 0.13 | 3.30×10^-4^ | 0.31 | 0.47 | -0.50 | 0.10 | 0.0084 | 3.78×10^-7^ | **1.36×10^-5^** |
| Left CST | -0.02 | 0.02 | 4.24×10^-4^ | 0.50 | 0.65 | 1.40×10^-3^ | 0.02 | 6.25×10^-6^ | 0.94 | 0.99 |
| Left SLF | 1.32×10^-3^ | 0.06 | 4.19×10^-7^ | 0.98 | 0.99 | -0.20 | 0.04 | 0.0208 | 2.51×10^-6^ | **4.52×10^-5^** |
| Left cingulate gyrus | -0.04 | 0.05 | 7.25×10^-4^ | 0.38 | 0.53 | -0.11 | 0.03 | 0.0104 | 8.99×10^-4^ | **3.64×10^-3^** |
| Left corona radiata | -0.03 | 0.12 | 4.50×10^-5^ | 0.83 | 0.93 | -0.32 | 0.08 | 0.0147 | 7.61×10^-5^ | **6.85×10^-4^** |
| Left external capsule | -0.21 | 0.06 | 0.0112 | 5.24×10^-4^ | **2.70×10^-3^** | -0.14 | 0.04 | 0.0118 | 3.96×10^-4^ | **2.37×10^-3^** |
| Left internal capsule | -0.02 | 0.08 | 3.18×10^-5^ | 0.85 | 0.93 | -0.21 | 0.06 | 0.0128 | 2.35×10^-4^ | **1.69×10^-3^** |
| Left posterior thalamic radiation | -0.08 | 0.05 | 0.0024 | 0.11 | 0.20 | -0.11 | 0.03 | 0.0096 | 1.35×10^-3^ | **4.40×10^-3^** |
| Left uncinate fasciculus | -0.01 | 0.01 | 0.0011 | 0.28 | 0.44 | 3.48×10^-3^ | 0.01 | 1.64×10^-4^ | 0.68 | 0.79 |
| Right CST | -0.02 | 0.03 | 8.23×10^-4^ | 0.35 | 0.51 | 1.93×10^-4^ | 0.02 | 1.17×10^-7^ | 0.99 | 0.99 |
| Right SLF | -0.15 | 0.06 | 0.0054 | 0.02 | **0.03** | -0.12 | 0.04 | 0.0074 | 5.05×10^-3^ | **0.01** |
| Right cingulate gyrus | -0.07 | 0.04 | 0.0023 | 0.12 | 0.21 | -0.07 | 0.03 | 0.0044 | 0.03 | 0.06 |
| Right corona radiata | -0.16 | 0.11 | 0.0019 | 0.15 | 0.25 | -0.20 | 0.07 | 0.0071 | 6.18×10^-3^ | **0.01** |
| Right external capsule | -0.13 | 0.05 | 0.0055 | 0.02 | **0.03** | -0.12 | 0.04 | 0.0096 | 1.47×10^-3^ | **4.40×10^-3^** |
| Right posterior thalamic radiation | -0.16 | 0.05 | 0.0101 | 9.10×10^-4^ | **3.64×10^-3^** | -0.08 | 0.03 | 0.0054 | 0.02 | **0.03** |
| Right uncinate fasciculus | -0.01 | 0.01 | 3.36×10^-4^ | 0.55 | 0.66 | -0.01 | 0.01 | 5.67×10^-4^ | 0.44 | 0.59 |
| **Molehills** |  |  |  |  |  |  |  |  |  |  |
| Cerebellar peduncle | 0.03 | 0.06 | 6.98×10^-5^ | 0.64 | 0.70 | 0.15 | 0.04 | 0.0041 | 3.82×10^-4^ | **2.29×10^-3^** |
| Corpus callosum | 0.39 | 0.12 | 0.0033 | 1.33×10^-3^ | **4.35×10^-3^** | 0.48 | 0.09 | 0.0095 | 6.71×10^-8^ | **1.21×10^-6^** |
| Left CST | -2.77×10^-3^ | 0.03 | 8.38×10^-6^ | 0.93 | 0.93 | 0.05 | 0.02 | 0.0047 | 0.03 | **0.04** |
| Left SLF | 0.16 | 0.07 | 0.0045 | 0.03 | **0.04** | 0.19 | 0.05 | 0.0136 | 1.50×10^-4^ | **1.35×10^-3^** |
| Left cingulate gyrus | -0.03 | 0.05 | 3.13×10^-4^ | 0.56 | 0.65 | 0.11 | 0.03 | 0.0092 | 1.74×10^-3^ | **5.21×10^-3^** |
| Left corona radiata | 0.36 | 0.11 | 0.0100 | 9.66×10^-4^ | **3.48×10^-3^** | 0.26 | 0.08 | 0.0111 | 6.16×10^-4^ | **3.17×10^-3^** |
| Left external capsule | 0.11 | 0.06 | 0.0026 | 0.09 | 0.12 | 0.15 | 0.05 | 0.0107 | 7.21×10^-4^ | **3.24×10^-3^** |
| Left internal capsule | 0.21 | 0.09 | 0.0046 | 0.03 | **0.04** | 0.17 | 0.06 | 0.0069 | 6.88×10^-3^ | **0.02** |
| Left posterior thalamic radiation | 0.01 | 0.05 | 5.05×10^-5^ | 0.81 | 0.87 | 0.13 | 0.04 | 0.0127 | 2.44×10^-3^ | **1.76×10^-3^** |
| Left uncinate fasciculus | -2.35×10^-3^ | 0.01 | 3.01×10^-5^ | 0.86 | 0.88 | 0.01 | 0.01 | 0.0017 | 0.18 | 0.22 |
| Right CST | -0.05 | 0.03 | 0.0030 | 0.07 | 0.11 | 0.06 | 0.02 | 0.0073 | 5.05×10^-3^ | **0.01** |
| Right SLF | 0.16 | 0.07 | 0.0045 | 0.03 | **0.04** | 0.15 | 0.05 | 0.0083 | 3.12×10^-3^ | **8.02×10^-3^** |
| Right cingulate gyrus | -0.14 | 0.05 | 0.0081 | 2.94×10^-3^ | **8.02×10^-3^** | 0.08 | 0.03 | 0.0054 | 0.02 | **0.03** |
| Right corona radiata | 0.06 | 0.12 | 3.01×10^-5^ | 0.58 | 0.66 | 0.27 | 0.08 | 0.0103 | 9.54×10^-4^ | **3.48×10^-3^** |
| Right external capsule | 0.11 | 0.07 | 0.0025 | 0.10 | 0.13 | 0.09 | 0.05 | 0.0035 | 0.05 | 0.08 |
| Right posterior thalamic radiation | 0.13 | 0.05 | 0.0054 | 0.02 | **0.03** | 0.09 | 0.04 | 0.0054 | 0.02 | **0.03** |
| Right uncinate fasciculus | 0.02 | 0.01 | 0.0017 | 0.18 | 0.22 | 5.00×10^-3^ | 0.01 | 3.21×10^-4^ | 0.56 | 0.65 |
| *Included: n=3,047 children, results were obtained by using multiple regression. Sex-specific effects were adjusted for age and total brain volume and age-specific effects were adjusted for sex, respectively. Lateralized tracts are additionally adjusted for handedness. Significant values after FDR multiple testing correction are highlighted in bold.*  *Abbreviations: beta (β), False Discovery Rate (FDR), p-value (p), standard error (SE), delta r-squared (ΔR^2^).* | | | | | | | | | | |

|  | **MS-PRS (P_T_<0.01)** | | | | |
| --- | --- | --- | --- | --- | --- |
| **Outcome, WM tract** | **β** | **SE** | **ΔR^2^** | **p** | **FDR** |
| **Molehills** |  |  |  |  |  |
| Cerebellar peduncle | 0.03 | 0.04 | 6.14×10^-4^ | 0.41 | 0.55 |
| Corpus callosum | 0.32 | 0.09 | 0.0120 | 2.54×10^-4^ | **4.57×10^-3^** |
| Left CST | 0.02 | 0.01 | 0.0023 | 0.11 | 0.24 |
| Left SLF | 0.04 | 0.03 | 0.0014 | 0.23 | 0.38 |
| Left cingulate gyrus | 0.01 | 0.02 | 3.65×10^-4^ | 0.53 | 0.64 |
| Left corona radiata | 0.09 | 0.05 | 0.0036 | 0.05 | 0.23 |
| Left external capsule | 0.07 | 0.03 | 0.0064 | 8.72×10^-3^ | 0.08 |
| Left internal capsule | 0.05 | 0.04 | 0.0013 | 0.23 | 0.38 |
| Left posterior thalamic radiation | 0.03 | 0.02 | 0.0023 | 0.12 | 0.24 |
| Left uncinate fasciculus | 0.01 | 0.01 | 8.60×10^-4^ | 0.34 | 0.51 |
| Right CST | -3.71×10^-3^ | 0.01 | 8.08×10^-5^ | 0.77 | 0.81 |
| Right SLF | 0.05 | 0.03 | 0.0024 | 0.11 | 0.24 |
| Right cingulate gyrus | 2.93×10^-3^ | 0.02 | 2.08×10^-5^ | 0.88 | 0.88 |
| Right corona radiata | 0.12 | 0.05 | 0.0054 | 0.02 | 0.10 |
| Right external capsule | 0.05 | 0.03 | 0.0026 | 0.09 | 0.24 |
| Right posterior thalamic radiation | -0.01 | 0.02 | 2.21×10^-4^ | 0.63 | 0.71 |
| Right uncinate fasciculus | 4.18×10^-3^ | 0.01 | 6.02×10^-4^ | 0.43 | 0.55 |
| *Included: n=1,087 children, data are corrected for age, sex and ten genetic principal components (PCs). Lateralized tracts are additionally adjusted for handedness. Significant values after FDR multiple testing correction are highlighted in bold.*  *Abbreviations: beta (β), corticospinal tract (CST), delta r-squared (ΔR^2^), False Discovery Rate (FDR), multiple sclerosis (MS), polygenic risk score (PRS), p-value (p), standard error (SE), superior longitudinal fasciculus (SLF), white matter (WM).* | | | | | |

**Supplementary Table 2: Effects of the MS-PRS (P_T_<0.01) on the number of molehills located in several WM tracts. (n=1,087)**

**Supplementary Table 3: Effects of the MS-PRS (P_T_<0.01) on the size of potholes and molehills at a cluster threshold of 50mm^3^. (n=1,087)**

|  | **MS-PRS (P_T_<0.01)** | | | | |
| --- | --- | --- | --- | --- | --- |
| **Outcome, size at 50mm^3^ cluster threshold** | **β** | **SE** | **ΔR^2^** | **p** | **FDR** |
| **Potholes** | -0.2 | 1.25 | 2.58×10^-5^ | 0.87 | 0.94 |
| **Molehills** | 0.1 | 0.85 | 5.15×10^-6^ | 0.94 | 0.94 |
| *Included: n=1,087 children, data are corrected for age, sex and ten genetic principal components (PCs). Significant values after FDR multiple testing correction are highlighted in bold.*  *Abbreviations: beta (β), False Discovery Rate (FDR), multiple sclerosis (MS), polygenic risk score (PRS), p-value (p), standard error (SE), delta r-squared (ΔR^2^).* | | | | | |

|  | **MS-PRS (P_T_<0.01)** | | | | |
| --- | --- | --- | --- | --- | --- |
| **Outcome, minimum voxel cluster size in mm^3^** | **β** | **SE** | **ΔR^2^** | **p** | **FDR** |
| **Molehills** |  |  |  |  |  |
| 25 | 3.0 | 1.29 | 0.0254 | 0.02 | **0.03** |
| 50 | 1.9 | 0.83 | 0.0241 | 0.03 | **0.03** |
| 100 | 1.5 | 0.54 | 0.0364 | 7.07×10^-3^ | **0.02** |
| 200 | 0.9 | 0.33 | 0.0365 | 8.04×10^-3^ | **0.02** |
| *Included: n=185 children, data are corrected for age, sex and ten genetic principal components (PCs). Significant values after FDR multiple testing correction are highlighted in bold.*  *Abbreviations: beta (β), False Discovery Rate (FDR), multiple sclerosis (MS), polygenic risk score (PRS), p-value (p), standard error (SE), delta r-squared (ΔR^2^).* | | | | | |

**Supplementary Table 4: Effects of the MS-PRS (P_T_<0.01) on the number of molehills in unique participants scanned in an earlier phase of the Generation R study. (n=185)**

**Supplementary Table 5: Effects of the MS-PRS (P_T_<0.01) on the number of molehills located in several WM tracts in the replication sample. (n=185)**

|  | **MS-PRS (P_T_<0.01)** | | | | |
| --- | --- | --- | --- | --- | --- |
| **Outcome, WM tract** | **β** | **SE** | **ΔR^2^** | **p** | **FDR** |
| **Molehills** |  |  |  |  |  |
| Cerebellar peduncle | 0.10 | 0.07 | 0.0122 | 0.14 | 0.38 |
| Corpus callosum | 0.14 | 0.08 | 0.0135 | 0.10 | 0.38 |
| Left CST | 0.01 | 0.01 | 0.0057 | 0.31 | 0.46 |
| Left SLF | 0.03 | 0.04 | 0.0024 | 0.49 | 0.62 |
| Left cingulate gyrus | -9.89×10^-4^ | 0.02 | 9.92×10^-6^ | 0.97 | 0.97 |
| Left corona radiata | 0.08 | 0.05 | 0.0116 | 0.14 | 0.38 |
| Left external capsule | 0.03 | 0.03 | 0.0083 | 0.21 | 0.38 |
| Left internal capsule | 0.05 | 0.05 | 0.0076 | 0.23 | 0.38 |
| Left posterior thalamic radiation | 0.05 | 0.03 | 0.0213 | 0.05 | 0.38 |
| Left uncinate fasciculus | 1.19×10^-3^ | 0.01 | 2.43×10^-4^ | 0.83 | 0.93 |
| Right CST | 0.01 | 0.01 | 0.0024 | 0.52 | 0.62 |
| Right SLF | 0.07 | 0.04 | 0.0150 | 0.09 | 0.38 |
| Right cingulate gyrus | 0.01 | 0.02 | 0.0023 | 0.52 | 0.62 |
| Right corona radiata | 0.08 | 0.06 | 0.0085 | 0.21 | 0.38 |
| Right external capsule | 0.04 | 0.03 | 0.0117 | 0.15 | 0.38 |
| Right posterior thalamic radiation | 0.03 | 0.02 | 0.0097 | 0.18 | 0.38 |
| Right uncinate fasciculus | 8.63×10^-4^ | 0.01 | 1.29×10^-4^ | 0.88 | 0.93 |
| *Included: n=185 children, data are corrected for age, sex and ten genetic principal components (PCs). Lateralized tracts are additionally adjusted for handedness. Significant values after FDR multiple testing correction are highlighted in bold.*  *Abbreviations: beta (β), corticospinal tract (CST), delta r-squared (ΔR^2^), False Discovery Rate (FDR), multiple sclerosis (MS), polygenic risk score (PRS), p-value (p), standard error (SE), superior longitudinal fasciculus (SLF), white matter (WM).* | | | | | |
