# Supplementary material for: White matter microstructural differences in children and genetic risk for multiple sclerosis: A population-based study
Source: Mult Scler. 2021 Aug 11;28(5):730–41. doi: 10.1177/13524585211034826 (PMC8978478; doi:10.1177/13524585211034826)
Supplement: sj-pdf-1-msj-10.1177_13524585211034826 – Supplemental material for White matter microstructural differences in children and genetic risk for multiple sclerosis: A population-based study [file sj-pdf-1-msj-10.1177_13524585211034826.pdf]

MS Journal Appendix for MRI methodology

Hardware	
Field strength	3T
Manufacturer	General Electric
Model	MR750w Discovery
Coil type<br>(e.g. head, surface)	Head coil
Number of coil channels	8 channels

Acquisition sequence		
Type<br>(e.g. FLAIR, DIR, DTI, fMRI)	DTI	
Acquisition time	8 min 8 sec	
Orientation	Axial	
Alignment<br>(e.g. anterior commissure/poster commissure line)	P/A	
Voxel size	2 mm^2	
TR	12,500 ms	
TE	72 ms	
TI		
Flip angle	90	
NEX		
Field of view	240 x 240 mm	
Matrix size	120 x 120	
Parallel imaging	Yes	No
If used, parallel imaging method:<br>(e.g. SENSE, GRAPPA)		
Cardiac gating	Yes	No
If used, cardiac gating method:<br>(e.g. PPU or ECG)		
Contrast enhancement	Yes	No

Acquisition sequence	
If used, provide name of contrast agent, dose and timing of scan post-contrast administration	
Other parameters:	

**Image analysis methods and outputs**	
Lesions	
Type<br>(e.g. Gd-enhancing, T2-hyperintense, T1-hypointense)	
Analysis method	
Analysis software	
Output measure<br>(e.g. count or volume [ml])	
Tissue volumes	
Type<br>(e.g. whole brain, grey matter, white matter, spinal cord)	
Analysis method	
Analysis software	
Output measure<br>(e.g. absolute tissue volume in ml, tissue volume as a fraction of intracranial volume, percentage change in tissue volumes)	
Tissue measures (e.g. MTR, DTI, T1-RT, T2-RT, T2*, T2', ^{1}H-MRS, perfusion, Na)	
Type<br>(e.g. whole brain, grey matter, white matter, spinal cord, normal-appearing grey matter or white matter)	Spatially independent clusters of aberrant WM structure
Analysis method	Pothole method (White et al. 2009)
Analysis software	FSL and Python
Output measure	Number of potholes and molehills globally and per tract
Other MRI measures (e.g. functional MRI)	
Type<br>(e.g. whole brain, grey matter, white matter, spinal cord, normal-appearing grey matter or white matter)	
Analysis method	
Analysis software	
Output measure	

Other analysis details:
